# President’s Address—Meeting of M. D. A.

**Published:** 1889-07

**Authors:** 


### Communications.

### President's Address.—Meeting of M. D. A., Grand Rapids, June 4th, 1889.

In bowing to the time honored custom of delivering the presiding officer's address before this association, it is with pleasure and pride that I am able to point to the progress that has been made in the last year in the arts and sciences pertaining to dentistry.

From what has been accomplished recently in microscopy in revealing the hidden forces of nature, through all the imperceptible gradations of birth, developement, life; and on the other hand the processes of retrograde metamorphosis, destruction, death; the dental profession preeminently has reason to congratulate herself upon the possession of so many skilled workers in this special field of study. The importance of the microscope in dentistry has never been felt so strongly as at present. The perfection of its machinery which enables a microscopist to confirm the truths of his patient research to a whole room full of people at once, is a wonderful step in advance.

Chemistry also has been sounded by our modern workers in its deepest waters.

The discovery and manufacture of many new medicaments has added important factors to our materia medica.

The recent classification of all our dental medicines according to their influence and action upon healthy and diseased fluids and tissues of the body, places our dental therapeutics upon a scientific basis never before attained.

And in like manner I might take up every branch of dentistry and find in it reasons for congratulating ourselves that we belong to such a progressive profession.

You have but to examine the dental periodicals, monographs and text books, issued since our last meeting of a year and three months ago, to become convinced of the large and important additions to our dental literature.

In mechanics and art also—in implements and materials, we have made great strides. And now, that that subtle force of nature—electricity—is fast becoming subservient to our commands, we can but see, in its various forms and methods, a power that is destined to revolutionize the possibilities of our resources.

Yet, as we advance as a profession, the field continues to broaden before us, and we hear the cry on every side for more knowledge of disease and its peculiarities, and something more complete and thorough to combat the fell destroyer.

Implements, materials, and methods of warfare are constantly changing like shifting sands of time. That which was the *ne plus ultra* yesterday, is bad form to-day. Yet hopefully, persistently, and more perfectly equipped, we press on to new fields to find the same old enemy in solid phalanx before us—clothed and accoutred by modern thought to be sure, yet, in reality, the same that stood before our daddies, and which they met—allowing them to tell the story—with about the same degree of success and failure.

So the question often arises:—Are we really making progress? Were the old methods of hand pressure, soft gold and saliva equal to our new fangled notions and present indispensables? or are our present failures largely due to the constantly increasing tendency of disease which keeps pace with our increasing knowledge and ability to combat it, as lock breakers keep pace with lock makers?

These are questions which each one of us must decide for ourselves. "Knowledge comes but wisdom lingers and she bears a laden breast filled with sad experience."

I believe that we do progress;—that the old methods, material, and hands, could not begin to do the work which we are accomplishing to-day. And tho' we have wallowed through many a mire, and been caught *en masse* in the delusive quick sands of unscientific and non-sensical things, we are fast shedding the last vestiges of empyracy and charlatanism. And while the ghost of that fossil of by-gone days still lurks in our rear, a being of more perfect proportions is proudly stepping to the front, armed with a knowledge of the laws and methods of nature from life to death--the scientific action of medicaments--materials and ways best adapted for restoration--and with hands ready to mould and shape according to correct principles of mechanism and art.

One of the evidences of our advancement, however unimportant it may be to us practically, is the willingness and even desire of the medical profession to extend to us the right hand of fellowship. And though this has awakened in us a variety of feelings and given rise to much controversy, still the fact must always remain that it was the act of a great fraternity bowing with respect and deference to the solid achievements we had attained as a distinct and separate body, and therefore one of which we should all be proud.

As I have said elsewhere "many seem proud and happy that we are at last officially recognized as something above mere tradesmen and mechanics, and allowed to shine even as a star of lesser magnitude in the horoscope of true professionalty; while others, seemingly with a more exalted opinion of unadulterated dentistry, and loyally sanguine of continued progress under the old flag—prefer to believe that "a formal acknowledgment of dentistry as a specialty of medicine," is of no importance specifically or essential to our professional dignity, and should be considered only as a compliment conferred upon a class of individuals possessing distinct characteristics, methods, literature and colleges of learning, between whom and the medical profession there is no chord of sympathy or interest other than is maintained between people of separate classes whose duties in life are in some particulars similar and demand occasional intermingling."

Whatever be the final decision we can but hope that the dental profession will preserve her integrity and be ready to give a united and hearty endorsement to that which is calculated to accomplish the greatest good to the whole.

Our phenomenal career is no doubt largely if not wholly due to the fact that we pull together as one man, working for the common good toward a higher standard of excellence. This we are enabled to do:—1. Through the co-operating influence of our national, state and local societies, which reveals the result of our every day work, that others may be benefited thereby; brings about the enactment of laws for the protection of ourselves and society; establishes codes for our guidance and the maintenance of professional dignity; destroys sectional jealousy and engenders good feeling and fellowship. 2. Through our periodicals, which are taking front rank as works of literary and scientific achievement, representative of a great and active profession; and as we feel here and there the pulse of these monthly heart throbs, we find them teeming with experience, research, and the best thoughts of toilers for the common good. 3. The interest and sympathy which we extend and ever maintain for our colleges of learning, constantly urging a higher standard of qualifications and a more thorough and complete course of didactic and practical training in every branch which pertains to dentistry, that men who come from them will not only be prepared to do dentistry, but qualified for an honorable place in society as gentlemen and scholars.

In these prime factors of our greatness and development we are par excellence compared to every other profession or fraternity. It is not strange therefore that many of our great men who have formed the nucleus of our comet-like progress should be unwilling for us to accept and hold a minor place as one of the elements of another fraternity when the future with all its possibilities so broadens before us as an independent profession.

Now, gentlemen of this association, what have the Michigan dentists been doing for the common good of our fraternity?

This State contains many dentists of no ordinary ability—men who are perfectly capable of contributing largely each year to our current dental literature and toward making this association compare favorably with other State societies. Yet the support which we give to these periodicals that are the very life of our progress, and to this society, is actually shameful compared to that which we might do if we were not so selfish and indifferent. It is so natural for a dentist after he has had years of active experience, and begins to get ripe with practical knowledge, to drift into a rut in which ease and comfort are the principal elements, and with a feeling that it is more blessed to receive than to give.

There is one thing, however, which we as Michigan dentists can point to with pride and pleasure:—The establishment of a dental college that has, through the influence of meritorious work, grown to rank second to none in the world. Around this we have centered our interests, our affections, and our aid—in fact the subject of our college, its needs and business pertaining thereto, have occupied so much of the time of this association in the past that the question has sometimes been asked if this society was sustained principally for the benefit and purposes of the college? And yet if there are no papers or clinics before us what better thing can occupy our time?

As I understand it, this association—like other State societies—is maintained for the elevation and advancement of dentistry, and for the education and benefit of its members. It therefore becomes the duty of every one who has the success of these yearly meetings at heart to do all that is possible to fill them to the brim with that which will prove a practical benefit to its members in their every day work. Then will its influence be felt throughout the State and prominent dentists from adjoining States be glad to partake of and add to its advantages.

Not many years ago I attended a meeting of this association at which there was only one paper read and no clinics given, the time being occupied principally in discussing and correcting the code of ethics, reprimanding and expelling infringers. While these things are important to us as an association it is not what the majority of us are here for, and therefore should occupy as little of our time as possible, and so far as is advisable put into the hands of committees, in order to be brought before this body in the most condensed form. The machinery of the society should not be clogged by long-winded interferences and every thing should be made subservient to questions pertaining to dentistry proper.

There are a number of recommendations which I would make relative to our constitution. I will therefore suggest that a committee of five be chosen to confer with me, and if thought advisable give notice of any change in contemplation to be brought before the next meeting.

At the last meeting of this association it was decided to change the time of our next annual meeting to May, it being a far pleasanter time of the year in Michigan, and on this account it was hoped that we would obtain a fuller attendance. Later the Board of Directors decided to again change the time to the first week in June so as to not interfere with the Illinois State Dental Association—believing it to be important that dentists in adjoining States should have the opportunity of attending each others meetings. In my opinion Michigan dentists can hardly afford to ignore the fact that we are near the great city of Chicago which alone contains over two hundred and seventy-five dentists, many of whom have won national reputations for great and useful contributions to the science and art of dentistry, and who, each year, give substantial aid to our progress. For a number of years this association has held its annual meetings in Detroit and Ann Arbor. It is hoped by breaking this regime and meeting at different places throughout the State our strength and numbers will be increased and a general interstate interest awakened in the advantages which these meetings afford.

We are therefore welcomed to the great and flourishing city of Grand Rapids with all the warmth and hospitality which its people and dentists can throw around us. Your Local Committee have spared no pains in seeing that we shall be well housed and fed, and all the advantages which the city affords for pleasure and profit freely opened to us. Your Executive Committee have succeeded in presenting a programme which they hope will prove interesting and instructive; and could I be sure that I shall discharge the duties of presiding officer of this association with proper ability, impartiality, and despatch, or even acceptably, I should feel a far greater assurance of the success of this meeting; for I assure you it is with the greatest embarrassment that I am forced to attempt that which I am so unfitted for in every way, knowing that I shall greatly tax your generosity and forbearance, and often require your most kindly aid.

Before closing it becomes my sad duty to announce the death of our most honored brother and fellow-worker, Dr. C. B. Porter, who died at his home in Bay City, March 20th. I would suggest that a committee be chosen to draft proper resolutions and condolences expressive of the sentiment of this association.

